# Jejunogastric intussusception after pancreaticoduodenectomy: a case report

**DOI:** 10.1186/s40792-022-01424-7

**Published:** 2022-05-10

**Authors:** Konosuke Yogo, Masanori Sando, Ryutaro Kobayashi, Genta Yano, Noriaki Ohara, Kiyotaka Kawai, Kenji Takagi, Satoru Kawai, Satoaki Kamiya

**Affiliations:** Department of Surgery, Tsushima City Hospital, 3-73, Tachibana-cho, Tsushima-City, Aichi 496-8537 Japan

**Keywords:** Intussusception, Pancreaticoduodenectomy, Child’s procedure, Complication

## Abstract

**Background:**

Jejunogastric intussusception (JGI) is a rare, but potentially fatal complication that can occur following gastric surgery, and the reported incidence of JGI is as low as 0.1%. Early diagnosis and treatment are critical for JGI to prevent major complications such as bowel necrosis and death. Although emergency surgery is the standard treatment, endoscopic reduction has also been reported to be effective in JGI patients without bowel necrosis. Several early recurrent cases treated with surgical or endoscopic reduction have been reported. We report an extremely rare case of JGI after pancreaticoduodenectomy (PD) using Child’s procedure that was successfully treated with surgical reduction and fixation.

**Case presentation:**

An 81-year-old man who had undergone PD using Child’s procedure 3 years ago presented to our hospital with epigastric pain and nausea. His vital signs were stable, and abdominal examination revealed mild tenderness with a palpable mass in the mid-epigastrium. Abdominal computed tomography (CT) and gastroscopy revealed a JGI of the efferent loop, and exploratory laparotomy was immediately performed. During the operation, the efferent loop showed no adhesions and was intussuscepted through the gastrojejunostomy into the gastric lumen. An incision in the anterior wall of the stomach revealed no evidence of ischemia of the intussusceptum. The efferent loop was reduced using Hutchinson’s maneuver and fixed to the afferent loop to prevent a recurrence. The postoperative course was uneventful, and there was no sign of recurrence 12 months postoperatively.

**Conclusions:**

JGI after PD is an extremely rare, but has severe complications. Surgery might be the optimal treatment for JGI in terms of preventing recurrence, even in cases without bowel necrosis.

## Background

Jejunogastric intussusception (JGI) is a rare but potentially fatal complication that can occur after gastrectomy or gastric bypass surgery. The reported incidence of JGI is as low as 0.1% [1]. Early diagnosis and treatment are critical for JGI to prevent major complications such as bowel necrosis and death. Although emergency surgery is the standard treatment, endoscopic reduction has also been reported to be effective in JGI patients without bowel necrosis [2]. However, several early recurrent cases treated with surgical or endoscopic reduction have been reported [3–6]. We report an extremely rare case of JGI after pancreaticoduodenectomy (PD) using the Child’s procedure that was successfully treated with surgical reduction and fixation.

## Case presentation

**Fig. 1:**
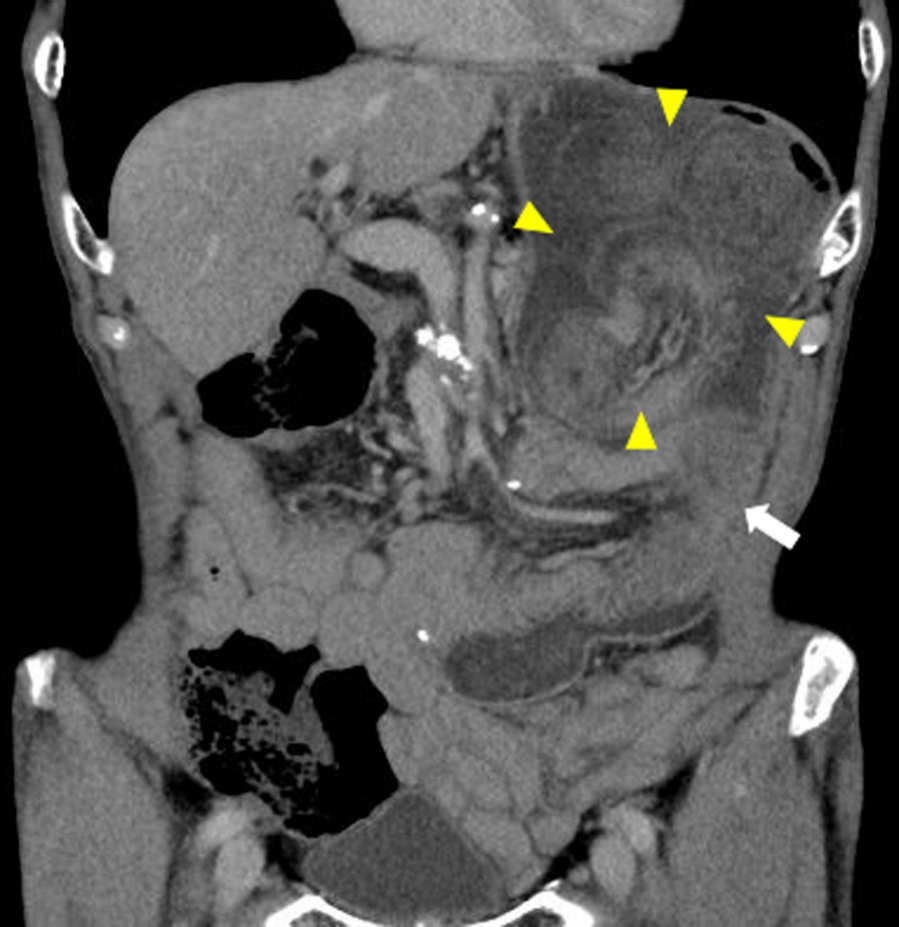
Contrast-enhanced computed tomography revealed that the small intestine (arrow), which had an edematous wall with poor contrast enhancement, had invaginated into the remnant stomach

**Fig. 2:**
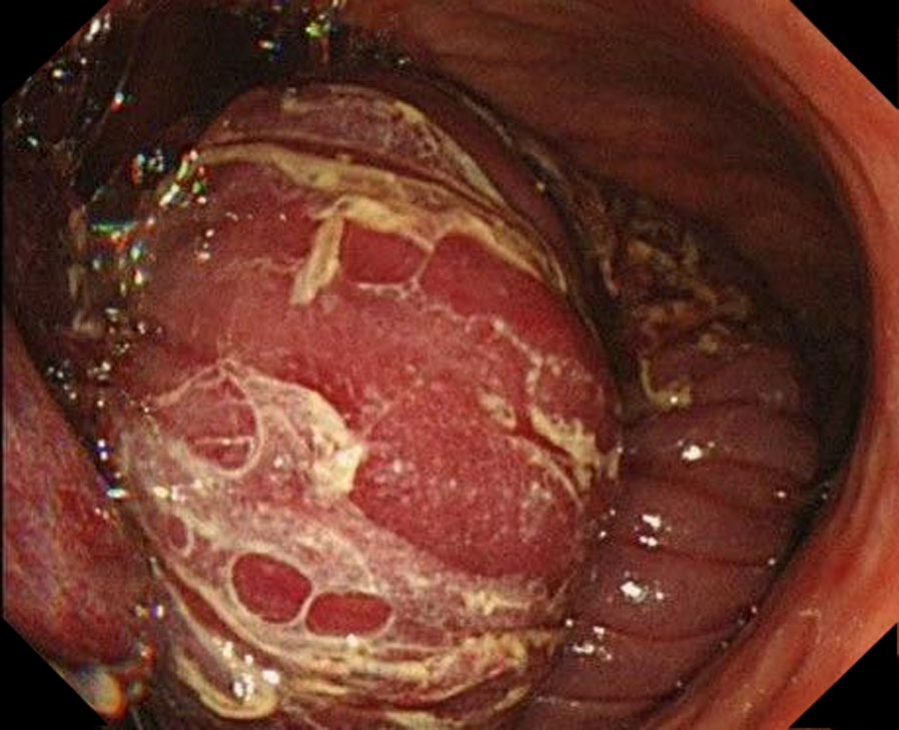
Gastroscopy revealed intussusception of the intestine into the stomach

**Fig. 3:**
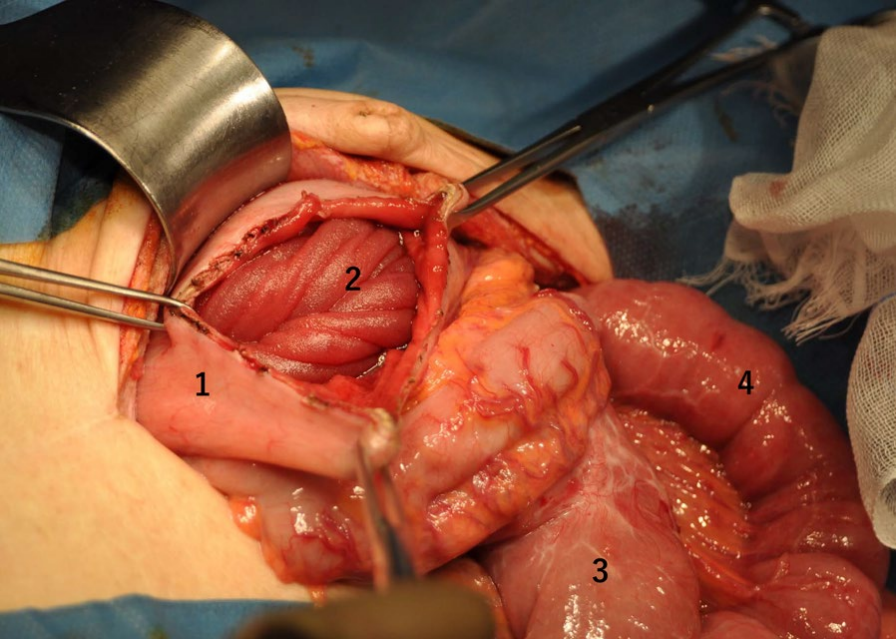
Intraoperative findings revealed jejunogastric intussusception. (1) Incised remnant stomach, (2) intussuscepted jejunum, (3) afferent loop, (4) efferent loop

An 81-year-old man with epigastric pain and nausea was admitted to our emergency department. He had undergone PD using Child’s procedure for distal bile duct cancer 3 years ago. Billroth II reconstruction by side-to-side retrocolic gastrojejunostomy without Braun’s anastomosis was used, and the remnant stomach was sutured to the mesenteric foramen. Additionally, enterostomy through the afferent loop was performed for postoperative nutritional management. His vital signs were stable, with a temperature of 36.4 °C, heart rate of 61 bpm, blood pressure of 150/83 mmHg, and SpO_2_ of 99% on room air. Abdominal examination revealed mild tenderness with a palpable mass in the mid-epigastrium. Laboratory examination results were unremarkable, except for a slight increase in the white blood cell count to 10,100/μL. Abdominal contrast-enhanced computed tomography (CT) revealed that the small intestine, which had an edematous wall with poor contrast enhancement, had invaginated into the remnant stomach (Fig. 1). Gastroscopy also revealed intussusception of the intestine into the stomach through a gastrojejunostomy (Fig. 2). CT and gastroscopy were suggestive of bowel ischemia; therefore, an exploratory laparotomy was immediately performed. During the operation, the efferent loop showed no adhesions and was intussuscepted into the gastric lumen using gastrojejunostomy. Making an incision in the anterior wall of the stomach enabled the reduction of the intussuscepted jejunum to that above 40 cm with Hutchinson’s maneuver, and the viability of the jejunal loop was preserved (Fig. 3). The incision was closed on the gastric wall. To prevent a recurrence, the efferent loop was fixed to the afferent loop that adhered to the abdominal wall because of a previous enterostomy. The postoperative course was uneventful, and the patient showed no recurrence 12 months after the surgery.

## Discussion

JGI was first described as a rare complication of gastroenterostomy by Bozzi in 1914 [7]. JGI after PD is an even rarer complication, and to the best of our knowledge, this is the fifth reported case [8–11]. Table 1 shows a summary of patients with JGI after PD, including our patient. Of the five cases, three occurred after Whipple’s procedure and the others after Child’s procedure, and the intussusceptums were efferent loops in four cases and afferent loops in the other. Emergency surgeries were performed in all cases, and the surgical procedures were reduction alone in one case, resection and reconstruction of the gastrojejunostomy in three cases, and reduction and fixation in our case. The time to intussusception from PD varied from 6 days to 5 years.

**Table 1:** Summary of patients with jejunogastric intussusception after pancreaticoduodenectomy

Author	Year	Age/sex	Diagnosis	Reconstruction	Antecolic or retrocolic	Braun’s anastomosis	Time after PD	Brynitz classification	Surgical procedure
Kishan	2019	37/M	P-NET	Whipple	N/M	N/M	9 months	Type 1	Resection and re-reconstruction
Yun-Xiao	2020	68/M	Ampullary cancer	Whipple	N/M	N/M	5 years	Type 2a	Reduction
Jiang-Jiao	2020	67/M	Pancreatic cancer	Child	Retrocolic	No	6 days	Type 2a	Resection and re-reconstruction
Moore	2020	68/F	Pancreatic cancer	Whipple	N/M	N/M	3 years	Type 2a	Resection and re-reconstruction
Our case	2022	81/M	Distal bile duct cancer	Child	Retrocolic	No	3 years	Type 2a	Reduction and fixation

*P-NET*, pancreatic neuroendocrine tumor, *PD* pancreaticoduodenectomy, *N/M* not mentioned

In the diagnosis of JGI, medical history of surgery and contrast-enhanced CT scan are essential. Alike previously reported [6, 12], the CT scan also showed that jejunal loop invaginated into remnant stomach accompanying its mesentery and mesenteric vessels. If patient’s general condition allows, gastroscopy is more useful to make an accurate diagnosis of JGI, revealing a so-called ‘green caterpillar-like’ lesion [13], a mass with mucosal folds protruding through anastomosis.

According to the intussusceptum, Brynitz classified JGI after Billroth II gastrectomy into five types [14]. Type 1 was afferent loop intussusception into the remnant stomach; type 2a, efferent loop intussusception into the remnant stomach; type 2b, efferent loop intussusception into the efferent loop itself; type 3, afferent and efferent loop intussusception into the remnant stomach; and type 4, intussusception at Braun’s anastomosis. The percentages were 5.5%, 70%, 6.5%, 10%, and 8%, respectively, with type 2a being most common. As gastrojejunostomy in PD resembles Billroth II gastrectomy, our case can be classified as type 2a, which is the most common JGI after Billroth II gastrectomy. A simple reason for the high ratio of type 2a is that the efferent loop is longer and more mobile than the afferent loop in Billroth II gastrectomy. Another possible reason is the fixation between the afferent loop and remnant stomach to prevent food reflux, which is also likely to prevent afferent loop intussusception; type 1. Contrarily, the risk of a type 1 JGI may increase in cases with long afferent loops. Furthermore, a gastrojejunostomy with Braun anastomosis might have reduced the risk of type 1 to 3 JGIs because the mobility of the afferent and efferent loops is restricted.

The etiology of JGI consists of mechanical factors associated with anastomosis and functional factors. Large anastomosis, postoperative adhesion, and abnormal peristalsis have been reported being possible causes of JGI [15–17], and retrograde peristalsis is suspected to be a major cause, especially in type 2a. Peristalsis of the small intestine is known to be induced by interstitial cells of Cajal in the duodenum [18]. Separation of the small intestine from the duodenum, as in Roux-en-Y gastrectomy, is considered a cause of abnormal peristalsis, including Roux stasis syndrome [19]. Similarly, the small intestine was separated from the duodenum in PD. Abnormal peristalsis might have appeared in the separated intestine, playing a part in causing JGI after PD. However, as aforementioned, type 2a is the most common form of JGI after Billroth II gastrectomies. Our hypothesis does not explain this finding alone. It appears appropriate to consider that multiple factors coincide with JGI.

The low incidence of JGI after PD would be attributed by the smaller number of surgeries itself compared to gastric surgery. Besides, high frequent complications after PD such as pancreatic fistula and intra-abdominal abscess could cause more adhesion. More adhesion links to less mobile jejunal loops, and therefore it could prevent intussusception. In our case, intraoperative findings revealed that the efferent loop was free of any adhesions, possibly because of the previous use of anti-adhesive material, and the anastomosis was not remarkably large. Owing to a lack of adhesion, retrograde peristalsis of the efferent loop might have caused the JGI in our case. Considering its high mobility of the efferent loop, adding Braun’s anastomosis or using Roux-en-Y reconstruction in the initial surgery might have prevented the JGI.

In cases of JGI with bowel necrosis, resection and reconstruction of the anastomosis will be necessary; however, in those where the viability of the involved bowel is maintained after reduction, the optimal treatment is yet to be discussed. Although there have been several case reports of endoscopic reduction [14], it is contraindicated because of its high risk of recurrence [3–5, 20]. Preferable treatment for JGI without bowel necrosis is a surgical intervention in terms of preventing recurrence. Fixing the reduced intestine to adjacent structures such as the stomach, colon, mesocolon, and falciform ligament of the liver was reported to be an effective procedure for preventing recurrences [21, 22]. In contrast, in some studies, resection and reconstruction of the gastrojejunostomy were advocated regardless of the viability of the intussusceptum [3, 23]. In our case, because the anastomosis was not excessively large, we decided that revision was unnecessary. Instead, we fixed the efferent loop to the afferent loop, which was fixed to the abdominal wall for enterostomy previously. Twelve months after the surgery, the patient was well without recurrence. Thus, it is critical to select a suitable surgical procedure according to the intraoperative findings.

## Conclusions

JGI after PD is an extremely rare but severe complication. Surgery might be the optimal treatment for JGI in terms of preventing recurrence, even in cases without bowel necrosis.

## Data Availability

Not applicable.

## Abbreviations

JGI: Jejunogastric intussusception
PD: Pancreaticoduodenectomy
CT: Computed tomography
